# Direct and Oxidative DNA Damage in a Group of Painters Exposed to VOCs: Dose – Response Relationship

**DOI:** 10.3389/fpubh.2020.00445

**Published:** 2020-08-19

**Authors:** Renata Sisto, Delia Cavallo, Cinzia Lucia Ursini, Anna Maria Fresegna, Aureliano Ciervo, Raffaele Maiello, Enrico Paci, Daniela Pigini, Monica Gherardi, Andrea Gordiani, Nunziata L'Episcopo, Giovanna Tranfo, Pasquale Capone, Damiano Carbonari, Barbara Balzani, Pieranna Chiarella

**Affiliations:** ^1^Department of Occupational and Environmental Medicine, Epidemiology and Hygiene, Italian Workers' Compensation Authority (INAIL), Rome, Italy; ^2^Department of Prevention, Prevention and Safety at Workplace, ASUR Marche, Ancona, Italy

**Keywords:** biological monitoring, genotoxicity, oxidative stress, urinary dose biomarkers, gene polymorphism, volatile organic compounds

## Abstract

Volatile organic compounds (VOCs) are present in several working activities. This work is aimed at comparing oxidative stress and DNA damage biomarkers to specific VOCs in the occupational exposure of painters. Dose-response relationships between biomarkers of oxidative stress and of dose were studied. Unmetabolized VOCs and their urinary metabolites were analyzed. Urinary Methylhyppuric acids (MHIPPs, xylenes metabolite), Phenylglyoxylic and Mandelic acid (PGA, MA ethylbenzene metabolites), S-Benzylmercapturic acid (SBMA, toluene metabolite), and S-Phenylmercapturic acid (SPMA, benzene metabolite) were quantified at the end of work-shift. Oxidative stress was determined by: urinary excretion of 8-oxodGuo, 8-oxoGua and 8-oxoGuo and direct/oxidative DNA damage in blood by Fpg-Comet assay. Multivariate linear regression models were used to assess statistical significance of the association between dose and effect biomarkers. The regressions were studied with and without the effect of *hOGG1* and *XRCC1* gene polymorphisms. Statistically significant associations were found between MHIPPs and both 8-oxoGuo and oxidative DNA damage effect biomarkers measured with the Comet assay. Oxidative DNA damage results significantly associated with airborne xylenes and toluene, whilst 8-oxodGuo was significantly related to urinary xylenes and toluene. Direct DNA damage was significantly associated to SBMA. *XRCC1* wild-type gene polymorphism was significantly associated with lower oxidative and total DNA damage with respect to heterozygous and mutant genotypes. The interpretation of the results requires some caution, as the different VOCs are all simultaneously present in the mixture and correlated among them.

## Introduction

Volatile Organic Compounds (VOCs) include a variety of chemicals present in many household products and used in several working activities. It is widely known that continuous exposure to these compounds is dangerous, as these substances have been classified as carcinogenic by IARC (1) with adverse effects on the human health. Because of the easy evaporation at room temperature, the organic solvents can spread easily in the environment and are toxic. Particularly in the occupational setting, VOCs must be handled following appropriate safety precautions such as wearing the personal protection equipment (PPE) in order to avoid excessive exposure.

A bibliometric analysis, published in 2019, examined the scientific literature published in the years 2016–2018 on VOCs' effect on human health. The investigators conclude that the most common diseases, potentially associated with VOCs, mainly involve the respiratory system, the blood system, and inflammation (2).

One of the main effects induced by VOCs on humans is the damage of nucleic acids causing oxidative stress, genotoxicity and inflammation (3, 4). Nucleic acid oxidative damage biomarkers are the first biomarkers indicating a potential risk of chronic diseases, including chronic obstructive pulmonary disease (COPD), as well as lung, bladder cancer and hematological malignancies (5).

The human biological monitoring of exposure consists in the determination of biomarkers, which can be dose biomarkers, measuring internal exposure levels to be compared with any (if known) biological limit value, effect biomarkers, which highlight early symptoms or dysfunctional situations still reversible with the improvement of the exposure situations, and susceptibility biomarkers, which express individual differences of genetic or acquired origin (6).

The biomonitoring of workers exposed to VOCs can provide a useful and early detection system for the initiation of cell dysregulation, which would help to prevent the development of disease (7).

Painters represent an important worker category exposed to VOCs and several studies investigating the potential effects of exposure to toluene, xylenes, ethylbenzene, styrene and paints in this worker population showed oxidative and genotoxic consequences (8–13). In particular, the study of Moro et al. (11) found induction of DNA damage evaluated by comet assay, but not of micronuclei, in industrial painters exposed to low toluene levels. An increase of oxidative DNA injury in occupational exposure to paint was reported by Chang et al. (14). The authors found in spray painters a significant correlation between urinary 8-hydroxydeoxyguanosine (8-OHdG) and exposure to ethylbenzene. The recent study of Londono-Velasco et al. (9) found that exposure of car painters to organic solvents and paints was associated to an increase of the oxidative damage to the DNA evaluated by Fpg comet assay on lymphocytes.

In the present study we analyzed in a group of 17 painters the exposure biomarkers indicating the oxidative stress on single base/nucleotide (8-oxoGuo, 8-oxodGuo, and 8-oxoGua) (15, 16) as well as the effect biomarkers as direct and oxidative DNA damage (17, 18). Since the metabolism of such compounds is carried out by specific enzymes, we took into account also the role of susceptibility biomarkers that were analyzed by genotyping (19). One is *hOGG1*, i.e., the human *hOGG1* gene encoding the 8-oxoguanine DNA glycosylase, whose activity is to catalyze the excision of the mutagenic lesion 7,8-dihydro-8-oxoguanine (8-oxoGua) from oxidatively damaged DNA. Sub-cellular location of this protein is in the nucleus and in the mitochondrion (https://www.uniprot.org/uniprot/O15527) (20). The other gene is *XRCC1* (X-ray repair cross-complementing protein 1), an enzyme involved in DNA single-strand break repair by mediating the assembly of DNA break repair protein complexes. *XRCC1* is an essential protein required for the maintenance of genomic stability as it is involved in DNA repair system. The main function of *XRCC1* is associated with its role in the single-strand break (SSB) and base excision repair (BER) pathways that share several enzymatic steps. *XRCC1* has a crucial role in the coordination of BER pathway and its interaction with *OGG1*, in modulating DNA reparative response, is reported (21). Both enzymes are responsible for the maintenance of the DNA integrity (22).

The objective of this work is to evaluate the association between dose and effect biomarkers, related to both oxidative stress and damage of nucleic acids in the exposure to relatively low dose of VOCs. In particular, the urinary concentrations of Methylhyppuric acids (MHIPPs, xylenes metabolite), Phenylglyoxylic and Mandelic acid (PGA, MA both ethylbenzene metabolites), S-Benzylmercapturic acid (SBMA, toluene metabolite) and S-Phenylmercapturic acid (SPMA, benzene metabolite) were quantified at the end of the work-shift. The oxidative stress was determined by means of different effect biomarkers, such as the direct oxidation products generated from the DNA and RNA repair and turnover as well as direct and oxidative DNA damage evaluated by Comet assay, with and without Fpg.

Multivariate linear regression models were used to assess the statistical significance of the association between dose and effect biomarkers. The regressions were studied with and without the effect of the *hOGG1* and *XRCC1* gene polymorphisms to find out the most susceptible genotypes in the repair of DNA damage. A major limitation of this study is related to the fact that several different VOCs are simultaneously present in the mixture. In order to evaluate how the different effect biomarkers are sensitive and specific with respect to each VOC, subjects exposure to each VOC should have been separately available. This ideal circumstance is typical of animal studies, and almost never occurs in occupational health field studies, especially when the low dose regime is what one is interested in. Keeping in mind its limitation, this study could prompt and represent a basis for further field studies with different exposure conditions and for controlled exposure animal studies.

## Materials and Methods

### Subjects and Study Design

For this work were enrolled 17 professional painters working in a naval industry in Central Italy.

During the same experimental campaign, other data were collected on the same workers. In particular audiological data, showing the hearing dysfunctionality in workers exposed to VOCs (23), and miRNA data were separately analyzed in other studies (24). The present study was formally approved by the local Ethic Committee of the Health local agency of the Region of Marche. The workers gave their informed consent in participating to the study. Two main working tasks have been identified, roller- and spray-painting, the latter being associated to a larger airborne concentration of aromatic solvents of the mixture. The workers used respirators with carbon filters as the potential exposure levels to VOCs are of the order of the Occupational Exposure Limits of the Italian legislation. All the subjects were male, two of them belonging to Caucasian ethnicity and the others to Bengalese ethnicity. The mean age was 39 years (range 21–54 years). Five workers were smokers. The exposure to solvents was assessed by personal air sampling and urine sampling performed before and after the work-shift. An anamnestic questionnaire was administered to the enrolled subjects under the researchers supervision, regarding the professional exposure to organic solvents but also about the personal lifestyle and habits, the general health status, the cigarette smoke and use of drugs. The experimental campaign was performed on June 25th, 2018.

### Personal Air Monitoring

The personal exposure to organic solvents was assessed by passive air sampling by means of Radiello® devices during the whole work-shift. Each Radiello was chemically extracted with carbon disulfide and the samples were analyzed by GC-MS (G1888A, coupled with a 6890N, AgilentTechnologies, Santa Clara, CA United States) equipped with a single quadrupole mass spectro-metric detector (5973 MSD System, Agilent Technologies) with the internal standard method for the target VOCs, namely ethyl acetate, benzene, toluene, ethylbenzene, p-xylene, m-xylene, o-xylene.

### Biological Monitoring

Unmetabolized VOCs in the urine were determined by GC-MS with the headspace analysis method (25). The concentrations of toluene, ethylbenzene, p-xylene, m-xylene, o-xylene excreted unchanged were measured in the urine samples at the beginning and the end of the work-shift. In the same samples, for each VOC, the concentration of its most common and specific urinary metabolite was also determined. These are 2, 3, and 4-methylhippuric acid (MHIPPs, metabolites of 2, 3, and 4-xylene), Phenylglyoxylic and Mandelic acid (PGA, MA both ethylbenzene metabolites), S-Benzylmercapturic acid (SBMA, toluene metabolite) and S-Phenylmercapturic acid (SPMA, benzene metabolite). The cotinine, the most specific metabolite of nicotine, as measured at the aim of quantifying the exposure to cigarette smoke.

All the metabolites have been determined by HPLC-MS/MS (Series 200 LC pump, PerkinElmer, Norwalk, CT, USA coupled with an API 4000 triple-quadrupole mass spectrometry detector, AB/Sciex, Ontario, Canada) equipped with a Turbo Ion Spray (TIS), in the urine samples of workers, both before and after the working shift. SPMA, cotinine, their deuterium-labeled internal standards and MA and PGA were determined following the method described in Tranfo et al. (26), and in Paci et al. (27), respectively. SBMA and MHIPPs were determined by suitably changing the method by Sabatini et al. (28).

The 8-oxoGua 8-oxodGuo, 8-oxoGuo and their internal standards were determined by following the method described in Andreoli et al. (29) with some modifications (15).

All the results were expressed as the ratio to the concentration of urinary creatinine, in order to normalize the results for the dilution grade of urine. Urinary creatinine was determined by the method of Jaffè using alkaline picrate test with UV/Vis detection at 490 nm (30). Samples with creatinine concentrations lower than 0.3 g/L or higher than 3.0 g/L were excluded from statistical analysis according to the American Conference of Governmental Industrial Hygienists (ACGIH) recommendation (31).

### Direct/Oxidative DNA Damage - Fpg Comet Assay

Specialized medical personnel collected, at end-shift of the third working day, whole venous blood samples from exposed workers by venipuncture in sterile heparinized disposable syringes and transferred them to the laboratory. We used (Fpg) modified Comet assay to measure direct and oxidative DNA damage. In particular, the Fpg is a glycosylase that recognizes and specifically cuts the oxidized bases (principally 8-oxoguanine) from DNA, producing apurinic sites converted in breaks by the associated AP-endonuclease activity detected by comet assay as Fpg sites estimating oxidative DNA damage. Lymphocytes were isolated on a Ficoll-based density gradient and suspended in 1 ml of PBS (without Ca^2+^ and Mg^2+^), then the procedure of Collins et al. (32), with minor modifications (33) was used. For each subject two GelBond films (1 to be treated with Fpg enzyme and the other without) with a first layer of Normal-Melting Agarose (NMA) 1% in PBS and a second layer of cell sample (a few thousand) in low-melting agarose (LMA) 0.7% in PBS, were prepared. It allows the detection of direct DNA lesions (single – double strand breaks and alkali-labile sites) and oxidative DNA damage, respectively. The slides with gel bond were bathed in lysis solution (2.5 M NaCl, 100 mM Na_2_EDTA, 10 mM Tris with 1% Triton X-100, and 10% DMSO added fresh) and kept in the dark for 1 h at 4°C. Then they were washed 3 times in enzyme buffer (50 mM Na_3_PO_4_, 10 mM EDTA, 100 mM NaCl, pH 7.5), drained and incubated with 50 ml of either buffer or Fpg (1 mg/ml in enzyme buffer) in the dark for 30 min at 37°C. The slides were placed in a horizontal gel electrophoresis tank filled with fresh alkaline buffer (1 mM Na_2_EDTA and 300 mM NaOH, pH 13) for 40 min at 4°C to allow denaturing and unwinding of the DNA and the expression of alkaline-labile sites. Electrophoresis was done in the same buffer at 25 V and 300 mA for 30 min to allow the fragments of damaged DNA to migrate toward the anode. The slides were then washed 3 times with Tris/HCl 0.4 M for 5 min and stained with 50 ml ethidium bromide (10 mg/ml). Slides were examined by eye at 200X magnification under a fluorescence microscope. Images of 100 randomly selected comets for each slide were acquired and analyzed with a specific image analyzer software (Delta Sistemi, Roma, Italy). For each subject we calculated the mean values of tail DNA%, tail length (TL), and tail moment (TM). Tail DNA% (ratio of intensity of the tail and total intensity of the comet) measures the number of broken pieces of DNA; TL (comet tail length) measures the smallest detectable size of migrating DNA (small DNA fragments with high capacity to migrate); TM (product of the tail length and the percentage DNA in tail) furnishes a measure of both the above parameters. We considered for each subject the mean value of Tail DNA%, TM and TL from enzyme-untreated cells to evaluate direct DNA damage.

Tail DNA%enz, TMenz, and TLenz from Fpg-enzyme treated cells evaluate total (direct and oxidative) DNA damage. We deducted tail DNA%, TM and TL from the tail DNAenz%, TMenz, and TLenz both in exposed and unexposed subjects to obtain oxidative DNA damage (Fpg sites).

The difference (tail DNAenz% - tail DNA%) was used to identify subject positive to oxidative DNA damage. In particular, subjects with mean values of the difference (tail DNAenz% - tail DNA%) exceeding a fixed arbitrary cut off value of 4 were considered positive to oxidative DNA damage.

### Genotyping

Genomic DNA was isolated from the whole blood of the workers by using the QiAmp DNA blood mini kit cat. N. 51306 (Qiagen, Germany) following the manufacturer's instructions. Polymerase Chain Reaction (PCR) of *hOGG1 Ser*^326^*Cys*, and *XRCC1 Arg*^399^*Gln* was performed in the thermocycler (Multigene optimax thermal cycler, Aurogene SRL, Italy). Each reaction mixture contained 1X PCR buffer, 100 ng of DNA, 1 Unit of Taq polymerase per DNA sample (Promega), 0.3 μM of Forward and Reverse primers, 0.2 mM of dNTP and 2 mM of MgCl_2_ in a total volume of 40 μl. Forward and reverse primers were purchased from Metabion GmbH (Germany-Dasit Carlo Erba-Italy). hOGG1 (F:ACTGTCACTAGTCTCACCAG, R:GGAAGGTGCTTGGGGAAT). Amplification conditions were: 95°C 7 min, 94°C 30,” 60°C 30,” 72°C 30,” 72°C 7 min. XRCC1 (F:TTGTGCTTTCTCTGTGTCCA, R:TCCTCCAGCCTTTTCTGATA). Amplification conditions were 95°C 7 min, 95°C 30,” 56°C 30,” 72°C 1 min, 72°C 7 min. PCR products were separated on 1–2% agarose gel with TBE buffer (Tris, Boric acid, EDTA) (Cat. BMR 918100 Euroclone MI, Italy), and stained with gel red solution (Biotium CA, US). For each polymorphic gene, 20 μL of each amplicon were digested in 1X digestion buffer (New England Biolabs, MA, US and Thermo Fisher Scientifics) undergoing enzyme inactivation according to the procedure suggested by the manufacturer‘s instructions. 15U of Fnu4HI enzyme were used for *hOGG1* digestion 2 h at 37°C with the following restriction pattern: wt (Ser/Ser) 200 bp; het (Ser/Cys) 200, 107, 100 bp; mut (Cys/Cys) 107–100 bp. 15 U of MspI were used for *XRCC1* digestion overnight at 37°C with the following restriction pattern: wt (Arg/Arg) 374 and 221 bp; het (Arg/Gln) 615, 374, 221 bp; mut (Gln/Gln) 615 bp. Twenty μL of digested products of each polymorphic gene were run on agarose gel, stained with gel red solution to verify each fragment length.

#### Statistical Analysis

Analyses were carried out with SPSS/PC statistical software package 19.0 (Inc., Chicago, IL, USA) and statistical software R (R Foundation for Statistical Computing, Vienna, Austria). The VOCs and their most important metabolites concentrations were measured as continuous variables. Normality of the distributions was assessed in according to the Kolmogorov–Smirnov tests. The significance level for all tests was *p* < 0.05 (two-tailed). Multivariate linear regression models were used to determine the statistical significance of the dose- response relationships in which the biomarkers of DNA damage, direct and oxidative, play the role of outcome variables and the dose biomarkers, both the unmetabolized VOCs and their metabolites, play the role of explanatory variables. The smoking habit and the age were introduced as confounders whilst the individual susceptibility, evaluated by means of the polymorphisms of the genes involved in detoxification, was considered among the explanatory variables. The gene polymorphism was treated as a three- or two-level factor. The fitted models were of the form lm (ox_stress_biomarker ~ dose_biomarker + polymorphism) (1).

## Results

The workers were all male, 15 of Bengali ethnicity, one from Tunisia and another one from Iraq. Their age ranged from 21 to 49 years, with a mean of 39 years. The characteristics of the workers' sample are reported in Table 1.

**Table 1:** Characteristic of the subjects with polymorphisms of the analyzed genes.

**Subject**	**Task**	**Smoke**	***hOGG1***	***XRCC1***
N1	Roller	NO	wt	wt
N2	Roller	YES	wt	wt
N3	Roller	NO	het	het
N5	Roller	NO	mut	het
N7	Roller	NO	het	mut
N10	Roller	NO	het	het
N11	Roller	NO	wt	mut
N12	Roller	YES	wt	wt
N13	Roller	NO	het	het
N15	Roller	na	wt	wt
N16	Roller	NO	wt	wt
N4	Spray	YES	wt	wt
N6	Spray	NO	wt	wt
N8	Spray	NO	mut	wt
N9	Spray	YES	wt	wt
N17	Spray	YES	mut	het
N18	Spray	NO	wt	het

*Wt, wild type; het, heterozygous; mut, homozygous mutant*.

### Personal Air Monitoring

The external dose in terms of personal VOCs vapor concentration averaged over the 17 exposed subjects resulted: acetone 1.32 mg/m^3^, ethyl acetate 7.28 mg/m^3^, benzene 0.014 mg/m^3^, toluene 34.1 mg/m^3^, n-buthyl acetate 1.19 mg/m^3^, ethylbenzene 12.9 mg/m^3^, p-xylene 13.3 mg/m^3^, m-xylene 33.0 mg/m^3^, and o-xylene 11.22 mg/m^3^. Each average value resulted well-below the corresponding Occupational Exposure Limit Value in Italy (OELV), whereas the potential exposure to the mixture was found on average close to half of the mixture limit value. This last is evaluated by normalizing the exposure level to each VOC to its TLV and summing up the contribution of all the VOCs in the mixture. This sum must not exceed unity.

### Biological Monitoring (Dose Biomarkers)

The urinary concentrations of the most specific VOCs metabolites are reported in Table 2. These values are all well-below the BEI (Biological Exposure Indexes) given by the ACGIH (400 mg/g Cr for the sum of PGA and MA, 25 μg/g Cr for the SPMA, 1.5 g/g Cr for the sum of the three MHIPPs and 300 μg/g Cr for the SBMA). In fact, although the exposure levels are quite close to the TLV for the mixture as regards the airborne concentration in the case of the spray painters, the personal protective equipment are able to reduce effectively the exposure. The VOCs' metabolites in Table 2 are relative to the end-shift sampling. The concentrations at the beginning and the end of the work shift are significantly different at the level *p* < 0.05.

**Table 2:** VOCs' metabolites concentrations in the end – shift urine.

	**MA (mg/g cr)**	**PGA (mg/g cr)**	**SPMA (μg/g cr)**	**2_MHIPP (mg/g cr)**	3 and 4_MHIPP^*^ (mg/g cr)	**SBMA (μg/g cr)**
Mean	7.33	4.40	1.73	12.33	57.31	14.70
Median	7.68	4.56	1.82	13.00	60.04	15.24
SD	8.09	4.75	1.66	13.73	63.21	15.40
5th perc	8.49	4.94	1.75	14.46	66.19	16.00
25th perc	8.50	4.81	1.87	14.69	65.85	15.90
75th perc	8.62	4.24	1.96	14.93	65.16	16.12
95th perc	8.94	4.42	2.08	15.67	67.13	16.67
Max	9.38	4.63	2.17	16.28	69.78	16.62
Min	7.93	4.30	1.81	13.44	65.49	16.55

* *The metabolites 3 and 4 methtylhyppuric acids are not separated by the chromatographic process. However, the occupational exposure limit value refer to the sum of the three metabolites*.

The mean urinary concentrations of the target unchanged VOCs measured at end of the work-shift, normalized to the creatinine concentration (μg/g cr), are reported in Table 3. On average, the maximum percentage increment between the beginning and the end of the work-shift occurred for the p-xylene, 22%, followed by the toluene, 14%.

**Table 3:** Urinary unchanged VOCs concentrations in the end – shift urine.

	**Ethyl acetate (μg/g cr)**	**Benzene (μg/g cr)**	**Toluene (μg/g cr)**	**n-butyl acetate (μg/g cr)**	**Ethylbenzene (μg/g cr)**	**p-xylene (μg/g cr)**	**m-xylene (μ g/g cr)**	**o-xylene(μg/g cr)**
Mean	463.9	20.9	62.0	6.1	32.2	27.6	101.9	56.4
Median	58.8	6.5	11.9	4.0	10.1	6.9	34.1	25.5
SD	1252.7	39.1	169.9	6.5	63.1	59.1	201.3	83.3
5th perc	27.8	2.5	6.0	2.4	3.3	1.0	13.0	13.1
25th perc	41.8	3.5	8.4	3.1	7.0	4.0	22.2	18.9
75th perc	103.3	17.6	16.4	6.5	15.8	8.3	48.7	39.4
95th perc	2505.0	73.4	271.9	15.6	160.8	168.1	496.4	223.6
Max	5015.3	163.2	688.5	29.5	242.6	205.8	788.0	333.2
Min	23.8	2.4	5.9	2.1	2.6	0.7	12.8	12.9

### Direct/Oxidative DNA Damage-Urinary Oxidized Bases (Effect Biomarkers)

In Table 4 the distribution of the direct DNA damage biomarkers (Tail DNA%, TM and TL) and of the oxidative stress biomarkers are reported. These last consist of the systemic oxidative DNA damage biomarkers (Fpg sites) and the urinary DNA and RNA oxidation products, 8-oxoGuo, 8-oxodGuo and 8-oxoGua. The biomarkers of total damage to the DNA are also listed in terms of TL, TM and Tail DNA% in presence of enzyme treatment. Fpg-comet assay also showed that 10 out of 17 studied workers (58.8%) were positive to oxidative DNA damage. Tail DNA% and comet oxidative DNA damage of this study are higher than those found for unexposed subjects in other studies by the same authors, in which the genotoxic and oxidative effects in exposed workers were compared to control groups (17). As regards the urinary oxidized nucleic acid bases, they provide useful information mainly because they are very sensitive to gradients in the exposure levels. Indeed, a significant difference was measured between the beginning and the end of the work-shift. Data from the same group of exposed workers are reported in Tranfo (15) in which these differences can be appreciated. In particular, the 8-oxodGuo quite doubles due to daily VOCs' exposure.

**Table 4:** Biomarkers of direct and oxidative damage to the DNA.

	**Direct DNA damage without Fpg enzyme**			**Total DNA damage direct and oxidative with Fpg enzyme**			**Oxidative DNA damage Fpg sites (enz-buff)**			**Urinary oxidized nucleic acid bases**		
	**Tail DNA %**	**TM (AU)**	**TL (μm)**	**Tail DNA % enz**	**TM enz**	**TL enz**	**Tail DNA %**	**TM**	**TL**	**8oxoGua (μg/g cr)**	**8-oxoGuo (μg/g cr)**	**8-oxodGuo (μg/g cr)**
Mean	17.68	5.67	26.85	22.39	7.87	37.40	4.71	2.20	10.55	11.21	16.13	5.53
Median	16.30	5.68	27.50	22.70	7.93	39.00	4.30	2.03	10.20	6.20	14.72	5.59
SD	4.35	1.76	7.05	4.39	1.48	7.12	3.07	0.95	5.16	13.69	6.12	1.90
5th perc	12.42	3.34	18.51	15.68	5.51	25.68	0.48	1.08	4.93	0.01	10.04	3.28
25th perc	15.30	4.22	20.71	18.90	7.60	33.30	2.10	1.53	5.70	0.83	11.20	4.05
75th perc	20.90	7.23	29.80	24.43	8.60	40.60	6.50	2.66	12.90	14.84	18.33	5.98
95th perc	24.42	8.09	35.76	28.60	9.83	44.96	9.6	3.70	19.55	32.53	28.30	8.17
Max	26.50	8.32	44.00	31.40	10.35	54.00	11.60	4.09	20.30	46.63	31.16	10.71
Min	10.50	2.94	14.80	15.60	5.17	24.40	0.40	0.58	3.69	0.01	9.94	2.99

*AU, arbitrary units*.

**Figure 1:**
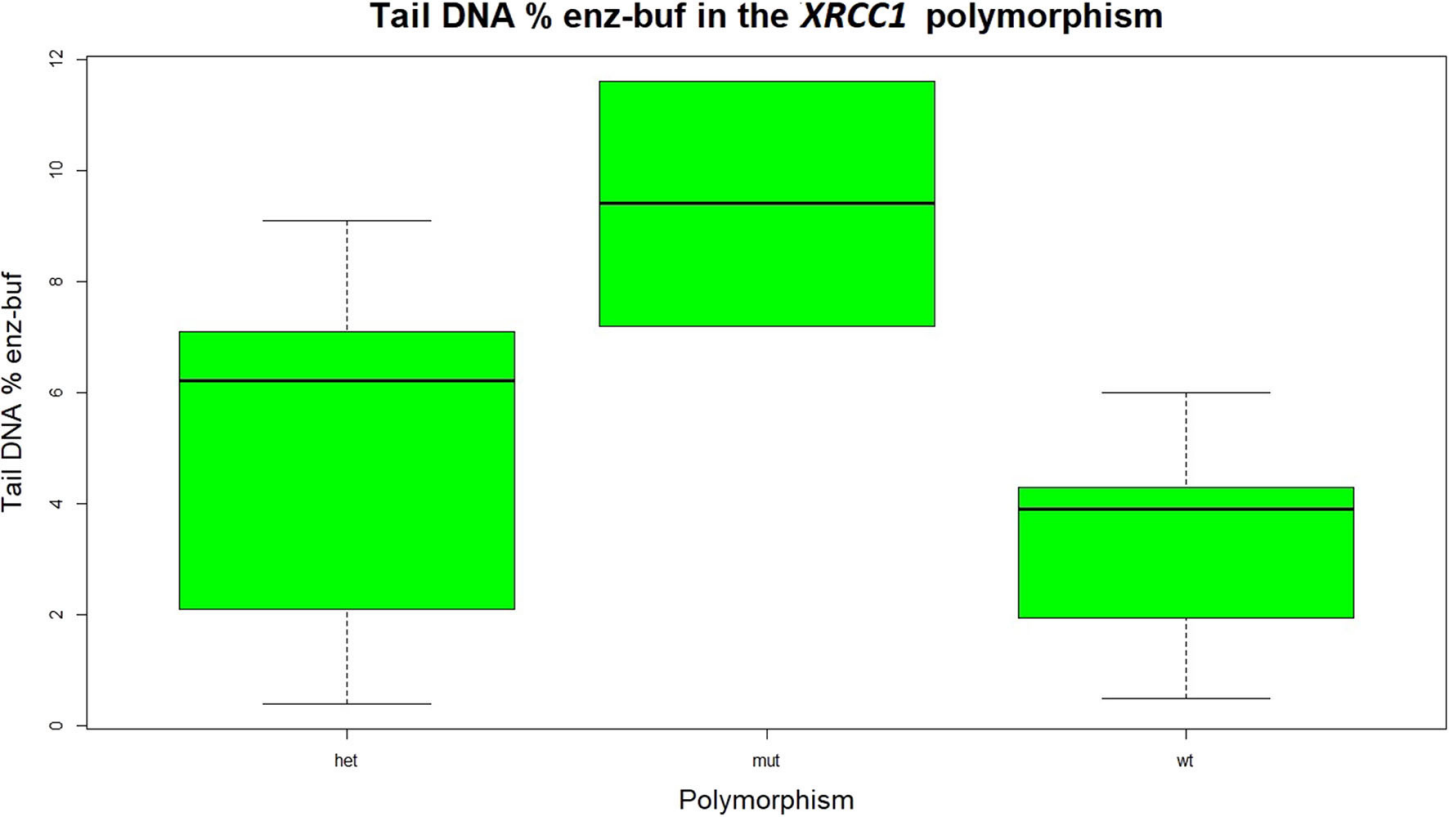
Statistical distribution of the DNA percentage difference, representing the oxidative damage to the DNA in the three different genotypes of the *XRCC1* gene. The wild type is significantly lower than the mutant genotype. The variant of the *XRCC1* with respect to the wild type are disadvantageous types, both the heterozygous and the mutant.

The effect of the polymorphisms on the oxidative stress biomarkers of the Comet test was studied by means of a one-way ANOVA. Two polymorphisms are differently expressed in the genotypes of our sample, i.e., *hOGG1* and *XRCC1*. The polymorphism of the *XRCC1* is significantly associated to the Tail DNA% enz-buf. The distribution of this variable as function of the three variants, wild-type, heterozygous and mutant is shown in Figure 1.

Tail DNA % enz-buf is lower in the wild type than in the heterozygous and mutant variants, both resulting disadvantageous genotypes. The difference between the heterozygous and the wild type was not statistically significant.

### VOCs Exposure-Oxidative Effect Relationship

As the dose biomarkers could be highly correlated among them due to the paint chemical composition, it is useful to analyze their correlation matrix, shown in Table 5.

**Table 5:** Correlation matrix of the dose biomarkers: VOCs metabolites measured in urine, airborne and urinary VOCs concentrations.

	**MA**	**PGA**	**SPMA**	**MHIPP**	**SBMA**	**Airborne ethylacetate**	**Airborne toluene**	**Airborne n_butil-acetate**	**Airborne ethyl benzene**	**Airborne xylenes**	**Urinary benzene**	**Urinary toluene**	**Urinary ethyl benzene**	**Urinary xylenes**
MA (μg/g cr)	1.00	0.63	0.73	*0.80*	0.15	0.37	0.31	0.03	0.48	0.53	0.69	0.35	0.51	0.53
PGA (μg/g cr)		1.00	0.32	0.66	0.07	0.20	0.16	−0.06	0.18	0.24	0.32	0.46	0.28	0.28
SPMA (μg/g cr)			1.00	0.72	0.15	0.53	0.59	−0.03	0.54	0.54	0.54	0.30	0.28	0.31
MHIPP (μg/g cr)				1.00	0.20	0.61	0.58	−0.09	0.71	0.73	0.51	0.55	0.54	0.56
SBMA (μg/g cr)					1.00	0.59	0.52	−0.29	0.42	0.38	0.23	−0.25	−0.18	−0.12
Ethylacetate (mg/m^3^)						1.00	*0.93*	−0.07	*0.88*	*0.87*	0.28	0.40	0.23	0.30
Toluene (mg/m^3^)							1.00	−0.17	*0.82*	*0.80*	0.02	0.45	0.09	0.15
n_butyl-acetate (mg/m^3^)								1.00	0.20	0.24	−0.04	0.69	*0.88*	*0.86*
Ethylbenzene (mg/m^3^)									1.00	*0.99*	0.27	0.75	0.59	0.62
Xylenes (mg/m^3^)										1.00	0.30	*0.80*	0.65	0.69
Benzene (μg/g cr)											1.00	−0.11	0.74	0.73
Toluene (μg/g cr)												1.00	*0.80*	*0.81*
Ethylbenzene (μg/g cr)													1.00	*0.99*
Xylenes_ (μg/g cr)														1.00

We notice that the correlation between the dose biomarkers belonging to different groups, i.e., VOCs metabolites, airborne and urinary VOCs concentrations is in general within 0.80 except in the case of the airborne n-butyl-acetate and the urinary ethylbenzene and xylenes concentrations, for which *r*^2^ = *0.88* and 0.86, respectively. The maximum correlation between the different metabolites is *r*^2^ = *0.80*, between MHIPP and MA. The airborne toluene is strongly correlated to the airborne ethylbenzene, *r*^2^ = *0.82* and to the airborne xylenes concentration, *r*^2^ = *0.80*. The urinary toluene concentration is strongly correlated to the urinary concentration of ethylbenzene and xylenes, *r*^2^ = *0.80* and *0.81*, respectively. The airborne and urinary concentrations of ethylbenzene are both highly correlated, *r*^2^ = *0.99*, to the respective concentrations of xylenes.

The significance of the association between the VOC's urinary concentrations and the VOC's metabolites and the oxidative biomarkers is shown in Table 6 in which the association between effect biomarkers and VOCs airborne concentration is also reported.

**Table 6:** Statistical association between the dose biomarkers and the biomarkers of oxidative stress.

**Oxidative stress biomarker**	**Dose biomarker**	**Model (1) without polymorphism**	**Model (1) with hOGG1 polymorphism**	**Model (1) with XRCC1 polymorphism**
TLenz – buffer (μm)	MHIPPs (μg/g cr)	*p* = 0.034	MHIPPs *p* = 0.028 *hOGG1 p* > 0.05	MHIPPs *p* = 0.04 *XRCC1 p* > 0.05
8-oxoGuo (μg/g cr)		*p* = 0.041	Ns	MHIPPs *p* = 0.04 *XRCC1 p* > 0.05
TLenz – buffer (μm)	Airborne xylenes (mg/m^3^)	*p* = 0.049	Air_xylenes *p* = 0.04 *hOGG1 p* > 0.05	Air_xylenes *p* = 0.019 *XRCC1 p* = 0.026
TLenz – buffer (μm)	Airborne toluene (mg/m^3^)	*p* = 0.006	Air_toluene *p* = 0.0017 *hOGG1 p* = 0.034	Air_toluene *p* = 0.0023 *XRCC1 p* = 0.056
TLenz – buffer (μm)	Airborne ethylbenzene (mg/m^3^)	*p* = 0.049	Air ethylbenzene *p* = 0.032 *hOGG1 p* > 0.05	Air_ethylbenzene *p* = 0.015 *XRCC1 p* = 0.02
TLenz – buffer (μm)	Airborne ethylacetate (mg/m^3^)	*p* = 0.023	Air ethylacetate *p* = 0.012 *hOGG1 p* > 0.05	Air ethylacetate *p* = 0.012 *XRCC1 p* > 0.05
8-oxodGuo (μg/g cr)	Urine p-xylene (μg/g cr)	*p* = 0.039	Ns	ns
8-oxodGuo (μg/g cr)	Urine toluene (μg/g cr)	*p* = 0.0018	Ns	ns

*The model (1) was fitted with or without the polymorphisms*.

The model given by equation (1) was fitted with and without the effect of the *XRCC1* or *hOGG1* polymorphism. The oxidative stress biomarkers are oxidative DNA damage in terms of TL, TM and Tail DNA% (Fpg sites enzyme-buffer differences) as it was explained previously and DNA turnover urinary biomarkers.

A significant association was found between the oxidative DNA damage (TLenz-buf) and the 8-oxoGuo and the MHIPPs concentration. The association between MHIPPs and TLenz-buf is strengthened if the polymorphism of the gene *hOGG1* is added. TLenz-buf is significantly associated to the airborne xylenes, toluene, ethylbenzene and ethyl-acetate concentrations and these associations are all strengthened if the polymorphisms of *hOGG1* and *XRCC1* are added.

The 8-oxodGuo is significantly (*p* = 0.039) associated to the xylenes concentration in urine and to the toluene concentration in urine (*p* = 0.0018). When the factor representing the polymorphism is significant, it indicates that the wild type is significantly associated to lower levels of oxidative stress biomarkers than the heterozygous and mutant variants that are not significantly distinguishable.

### VOCs Exposure-Direct DNA Damage Relationship

The direct damage to the DNA was evaluated by means of the genotoxicity biomarkers coming from the Comet test, i.e., TM, TL, and Tail DNA%. TM and the Tail DNA% were both significantly associated to the SBMA, urinary toluene metabolite. TL is associated to the toluene concentration in urine. The results of fitting the model (1) without the polymorphisms are shown in Table 7.

**Table 7:** Statistical association between dose biomarkers and biomarkers of direct DNA damage.

**Direct DNA damage biomarker**	**Dose biomarker**	***p*-value**	**β coefficient**	**Standard error**
TM	SBMA (μg/g cr)	0.045	0.13	0.06
Tail DNA %		0.023	0.36	0.14
TL (μm)	Urine toluene (μg/g cr)	0.037	−0.022	0.009

The biomarkers of direct damage to the DNA were found not significantly associated to the polymorphism of the *hOGG1* and *XRCC1*. In Table 7 the β coefficient of the regressions, the increment of the effect biomarkers when the dose biomarker is incremented by a unit, and its standard error are also shown.

**Figure 2:**
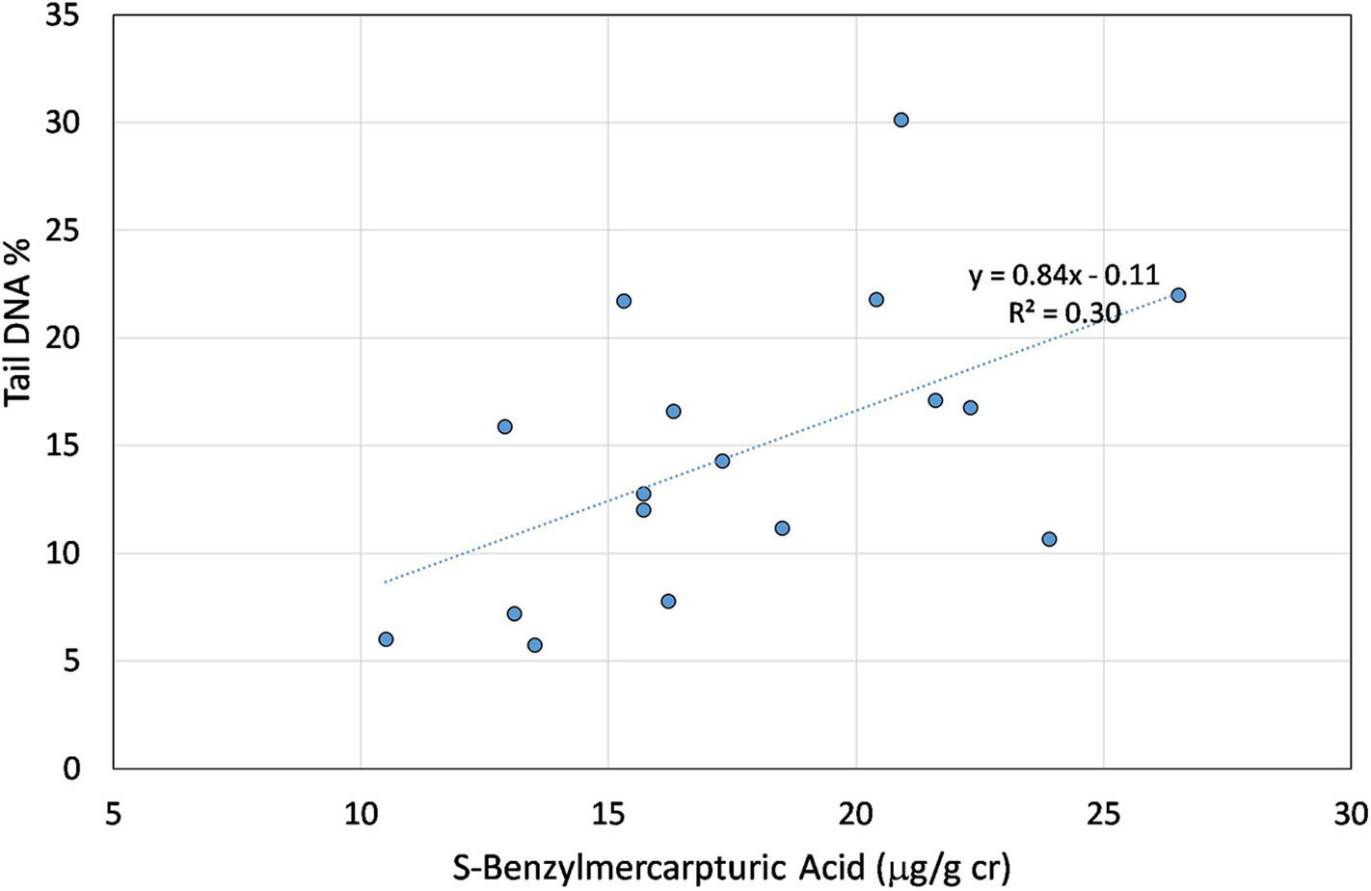
Linear association between the S-Benzylmercapturic acid concentration and percentage of the DNA in the tail used as biomarker of direct damage to the DNA.

The linear regression of the DNA percentage in the tail and the SBMA is shown in Figure 2.

The regression is statistically significant and its R2 is 0.30. The VOCs' metabolites of this study are much higher than those found in smokers as regards the MA, PGA, MHIPPs (34). Differently SPMA and SBMA are about one half the average concentrations found by Lorkiewicz in a population of smokers. However, the effect of the smoking habit in the associations between the direct DNA damage and the SBMA was not significant. This result is confirmed by the fact that the cotinine is not significantly associated to the biomarkers of direct damage to the DNA.

### VOC Exposure-Total (Direct and Oxidative) DNA Damage Relationship

In Table 8 the significance of the associations between the biomarkers of total damage to the DNA, i.e., the tail length, tail moment and DNA percentage in presence of the Fpg digestion enzyme, and the dose biomarkers are listed.

**Table 8:** Statistical association between dose biomarkers and biomarkers of total (direct and oxidative) damage to the DNA.

**Total DNA damage biomarker**	**Dose biomarker**	**Model (1) without polymorphism**	**Model (1) with XRCC1 polymorphism**
TLenz (μm)	MHIPPs (μg/g cr)	ns	MHIPPs *p* = 0.049 *XRCC1 p* = 0.05
TMenz	SBMA (μg/g cr)	ns	SBMA *p* = 0.039 *XRCC1 p* = 0.007
Tail DNA% enz		*p* = 0.025	SBMA *p* = 0.015 *XRCC1 p* > 0.05

*Only the significant relations are reported. The model (1) was fitted with and without the polymorphism of the gene XRCC1*.

Tail DNA % enz is significantly associated to the SBMA, this regression seems to be dominated by the direct effect. In the case of the TLenz the association with MHIPPs becomes significant when the polymorphism of the *XRCC1* is added to the model. The TMenz is significantly associate to the SBMA if the *XRCC1* polymorphism is kept into account into the model.

**Figure 3:**
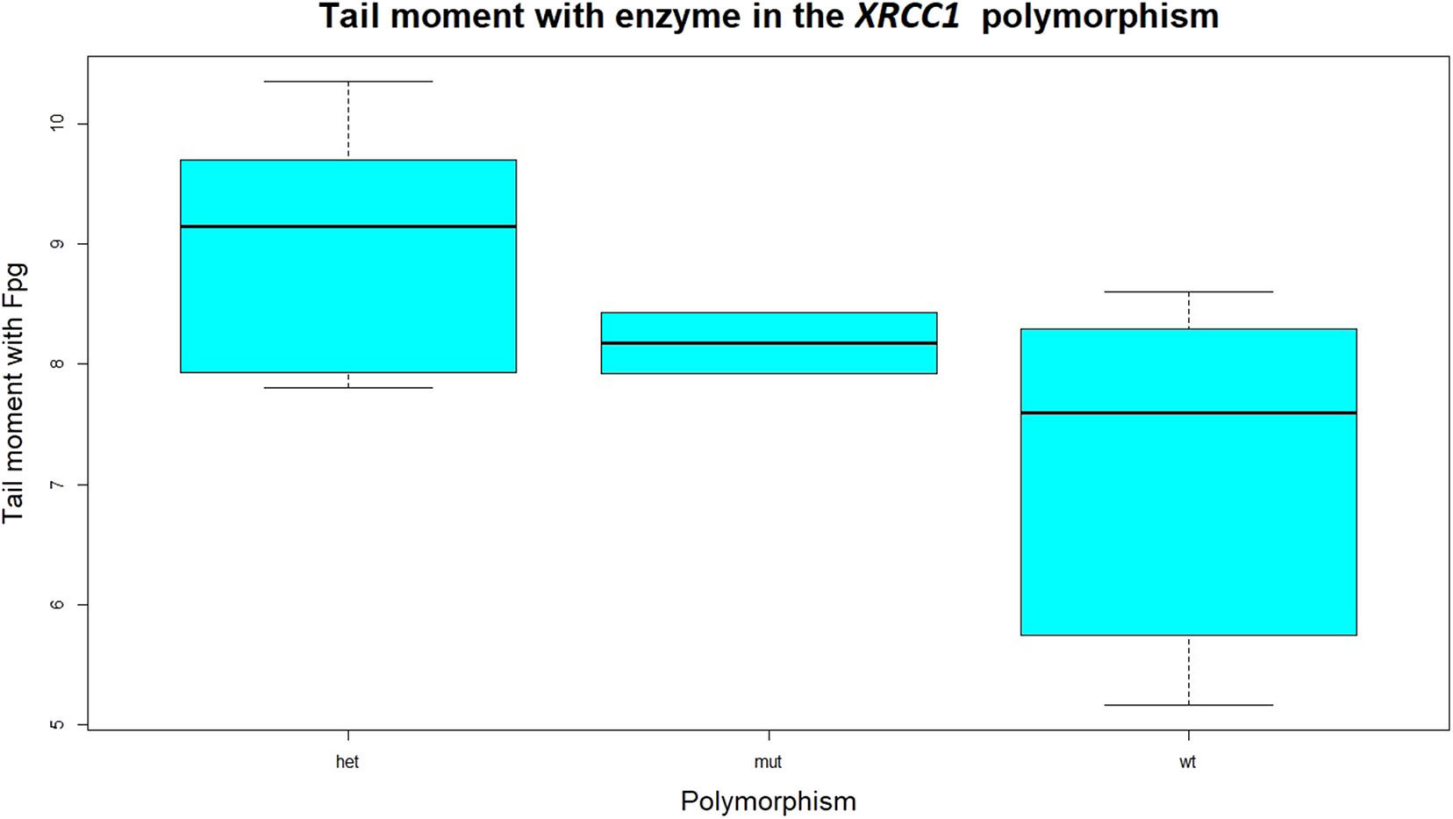
Statistical distribution of the tail moment with enzyme, representing the total DNA damage, direct and oxidative in the three different genotypes of the *XRCC1* gene. The wild type is significantly lower than the heterozygous and the mutant genotypes. The difference between mutant and heterozygous is not statistically significant. The variant of the *XRCC1* with respect to the wild type are disadvantageous types, both the heterozygous and the mutant.

A statistically significant association was found between the total DNA damage biomarker TMenz and the polymorphism of the gene *XRCC1* with the wild type significantly (*p* = 0.026) lower than the heterozygous (Figure 3).

**Figure 4:**
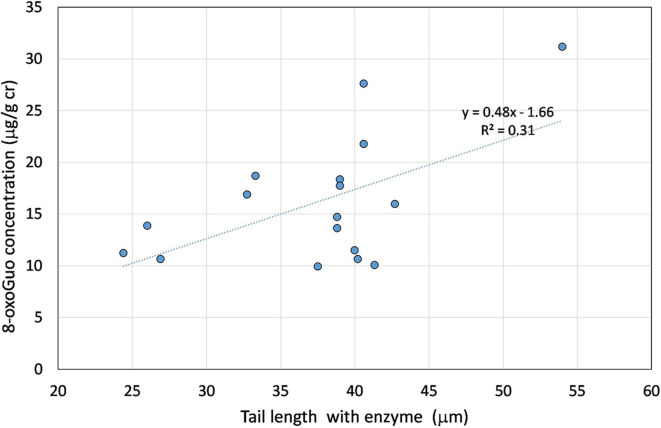
Linear association between the tail length in presence of the formamidopyrimidine glycosylase enzyme and the 8-oxoGuo concentration. The statistical association between the two variables is statistically significant (*p* = 0.042).

The association between the comet assay total DNA damage biomarkers and the urinary nucleic acid oxidation biomarkers were studied. A significant (*p* = 0.042) association was found between the 8-oxoGuo concentration, representing the RNA oxidation products at the end of the metabolic path, coming from the RNA turnover, and the Comet test tail length in presence of the digestion enzyme. This last permits the evaluation of the oxidation processes. In fact, the digestion enzymes transform the damage to breaks so amplifying the DNA quantity in the tail of the comet test. The oxidation products of the purines and pyrimidines are transformed in DNA breaks by the digestion enzymes, endonuclease III and Fpg, respectively (35). The association between the biomarker of total, direct and oxidative damage to the DNA, and the 8-oxoGuo is shown in Figure 4.

Similar significant associations were found between the TLenz and the 8-oxoGuo and the 8-oxoGua evaluated at the beginning of the work-shift.

## Discussion

In the occupational setting, such as in the naval ship painting, professional workers are daily exposed to different mixtures of VOCs which are extremely toxic and carcinogenic. Although the risk of inhalation and dermal contact is strictly controlled by the use of suitable PPEs, a minimal amount of these compounds may be absorbed and metabolized by the body enzymes, resulting in generalized toxicity and damage of nucleic acids. The DNA lesions may occur particularly on the guanine, the nitrogen base most sensitive to oxidation and mutagenicity (16).

The main objective of the present work was to find out quantitative relations between the dose biomarkers and the biomarkers of damage, direct or oxidative or both, to the DNA. In order to do that we designed dose–response curves, in which the effect biomarkers are considered as outcome variables and the dose biomarkers as explanatory ones. The dose–response relationships permit to quantitatively evaluate a very early risk curve with respect to genotoxic damage. As regards the oxidative stress both oxidative DNA damage and nucleic acid oxidation products at the end of the metabolic path, 8-oxoGuo and 8-oxodGuo, were kept into account.

Significant associations were found between oxidative DNA damage in terms of Tail length (the most sensible comet parameter to show the number of generated fragments in the case of low/moderate DNA oxidation), the 8-oxoGuo and the Methylippuric acids concentration (*p* = 0.034). This last are specific metabolite of xylenes, which represent one of the most important components of the mixture that workers are exposed to, the other one being the toluene. The 8-oxodGuo was also found significantly associated to the Methylippuric acids. The 8-oxodGuo, coming from the DNA repair and turnover, was also found significantly associated to the urinary concentrations of both xylenes and toluene. The association between MHIPPs and oxidative DNA damage (TLenz-buf) is strengthened if the polymorphism of the gene *hOGG1* is added. Higher level of urinary biomarkers of nucleic acid oxidation in particular 8-oxoGuo, correlating with internal exposure metabolites MA+PGA, have reported by Manini et al. (36) in styrene-exposed workers employed in two plastic lamination plants in comparison to controls. The authors concluded that styrene exposure seems to be associated with oxidation to nucleic acids, particularly to RNA and with induction of basic excision repair (BER) system (36). RNA is single-stranded and its bases aren't protected by hydrogen bonds or structural proteins and may be more susceptible to oxidative insults than DNA (37). Moreover, the presumable intra-cytoplasmatic ROS increase related to VOC induced oxidative stress is compatible with secondary oxidation of RNA molecules located in the cytoplasm and it could explain the 8-oxoGuo excretion associated with xylene exposure.

As the xylenes, toluene, ethylbenzene and ethylacetate airborne concentrations are strongly correlated, *r*^2^ > = *0.80*, the causal implications of the statistical associations with TLenz-buf reported in Table 6 cannot be univocally assessed. In other words, only one or two of the above-mentioned chemicals could be causally related to the DNA oxidative damage effect, while the others could just be correlated due to the paint composition. On the other hand, the observation that only the xylenes urinary metabolite (MHIPP) is correlated with TLenz-buf suggests that the causal association likely involves the xylenes, while for the other chemicals no conclusion can be drawn. Similarly, the correlations with the 8-oxodGuo of the strongly correlated toluene and xylenes urinary concentrations, cannot be attributed to one or the other chemical, but the relation of 8-oxodGuo with DNA repair mechanisms suggests that the main causal role is probably attributable to the toluene, whose metabolite SBMA was found associated to a direct damage to the DNA (Table 7).

Definitely our findings confirm the induction of oxidative nucleic acid damage in association with xylene and toluene exposure found either in blood and urine.

All data achieved during the biomonitoring campaign take into account the contribution of specific polymorphic enzymes, which are involved in the metabolism of the chemical compounds with different efficacy and depending on their genetic background. In particular, we took into account the *hOGG1 Ser326Cys* and *XRCC1 Arg399Gln* polymorphic genes, since they are relevant for the maintenance of the DNA stability (38, 39). The polymorphism as unique factor was significant only in the case of the *XRCC1* gene. Statistical significant associations were found between the *XRCC1* polymorphism and oxidative DNA damage in terms of Tail DNA % enz-buf. The *XRCC1* was also significant in explaining the TMenz related to the total, direct and oxidative damage to the DNA. The presence of the polymorphism as second factor affecting the dose-response relationships often strengthens the significance of the association between the dose and the effect biomarkers. In general, the wild type genotype of both the *XRCC1* and *hOGG1* was found significantly associated to lower levels of damage to the DNA biomarkers, the heterozygous and mutant genotypes being not significantly distinguishable. The conclusion is that the variants, with respect to the wild-type, of both *XRCC1* and *hOGG1* are disadvantageous, meaning that subjects with wild-type genotype (53% of the workers in the case of *XRCC1*) have more functional DNA repair ability compared to the other two genotypes, whose capability in the maintenance of DNA stability is less efficient. The *XRCC1* and *hOGG1* polymorphisms can be considered as susceptibility indices that could be kept into account in the occupational risk evaluation.

In addition to the oxidative DNA damage, the direct one was kept in consideration. A significant association was found between Tail DNA% and TM biomarkers with SBMA, specific metabolite of the toluene, while the TL biomarker was significantly associated to the urinary toluene concentration. Direct DNA damage by comet assay in painters exposed to relatively low toluene levels was also found, together with lipid peroxidation, by Moro et al. (11). The same authors showed in a previous study increased levels of oxidative stress biomarkers Malondialdehyde (MDA), superoxide dismutase (SOD) and Catalase (CAT) in industrial painters exposed to toluene, xylene, styrene, ethylbenzene and lead suggesting toluene as the principal factor responsible for increased lipid peroxidation (10).

One interesting finding of our work is that the toluene is mainly associated to the biomarkers of direct damage in the Comet test whilst the xylenes are significantly associated to the biomarkers of oxidative stress. As regards the associations between the urinary oxidative stress biomarkers and those coming from the Comet test, a significant relation was also found between the TLenz, representing a total DNA damage, and the 8-oxoGuo, particularly related to the RNA turnover and repair. Some caution must be taken in interpreting the level of specificity of the dose response relations, as a multicollinearity problem occurs between the different VOCs, In addition, it is not possible to exclude an interaction, possibly synergistic, between the different solvents in inducing an oxidative or direct damage to the DNA. Although this limitation should always be considered, this research could represent a useful paradigm for further investigations, to find out useful associations between solvent exposure and different dose and effect biomarkers, contributing in the mitigation of the health risk in the occupational setting.

## Conclusions

In this study, specific dose–response relationships are proposed, which permit the quantitative evaluation of very early risk curves with respect to genotoxic damage in workers exposed to a mixture of VOCs. Such curves relate dose biomarkers, i.e., unchanged VOCs and their metabolites, to direct or oxidative damage to the DNA. This study suffers from the main limitation that the different VOCs are all correlated among them. As consequence, the results should be interpreted with caution, because the correlation between the different VOCs does not permit a rigorous evaluation of each separate effect. The dose-response relations found in this study hold in a range of mild VOCs exposure, showing that, also in this range, some damage to the DNA, both direct and oxidative, does occur. Occupational exposure to chemical agents has been demonstrated to produce a measurable level of oxidatively generated damage to DNA and RNA, which is repairable only in the case of DNA. Effects of workplace exposure to asbestos, benzene, fine particulate matter, polycyclic aromatic hydrocarbons, silica, metals, styrene, toluene, and xylenes on the level of urinary 8-oxodGuo have been reported in the literature.

Even in conditions which are regarded as not dangerous, below the occupational exposure limit values, there is a detectable increase in the biomarkers concentration after a working shift, even if this is still within the range measured in the general population (15).

Early effect dose–response relations are crucial at the aim of preventing diseases resulting from a long-term low–dose exposure to VOCs. In more detail, linear regression models showed significant associations between oxidative stress biomarkers, i.e., oxidative DNA damage (TLenz-buffer difference), the 8-oxoGuo urinary concentration, and the Methylhippuric acids, specific metabolite of the xylenes. Oxidative DNA damage in terms of TL was found also significantly associated to the airborne xylenes, toluene, ethylbenzene and ethylacetate concentrations, whilst the 8-oxodGuo concentration was significantly associated to the urinary concentration of both xylenes and toluene. These results confirm that oxidative DNA damage by Fpg-comet and both urinary 8-oxodGuo and 8-oxoGuo represent valuable biomarkers for the biomonitoring of occupational exposure to VOCs, also at low concentration levels. In particular, these findings seem to suggest that 8-oxoGuo, related to RNA oxidation, and TL are the most sensitive biomarkers to evaluate early and still reparable oxidative effects of occupational exposure in painters, even at levels well-below the Biological Exposure Indexes.

In the case of the biomarkers of direct damage to the DNA, significant associations were found between Tail DNA%, TM and the SBMA toluene metabolite, and between TL and urinary concentration of toluene.

The study of the polymorphism of the XRCC1 gene on the direct and oxidative damage to the DNA showed that the oxidative DNA damage in terms of tail DNA% in the wild type is significantly lower (meaning a lower level of oxidative stress) than in the other two genotypes, heterozygous and mutant. As regards the total (direct plus oxidative) damage to the DNA, TMenz was found significantly lower in the wild type than in the other two genotypes. The wild type of *XRCC1* seems to be associated to lower levels of damage to the DNA being the heterozygous and the mutant both disadvantaged and not significantly different between them. The *hOGG1* was found not significantly associated to the DNA damage biomarkers when it is considered as the only factor. When multivariate mixed effects regressions are considered in which the polymorphisms are added to the dose biomarkers, the effect of the *hOGG1* polymorphism becomes significant, in the association between oxidative DNA damage in terms of TL and the airborne toluene.

As regards the polymorphism of *XRCC1* when it is added to the dose biomarkers it becomes significant in the case of the total DNA damage biomarkers and in particular in the associations between TMenz, TLenz, and MHIPPs or SBMA. In general, the polymorphisms of both *XRCC1* and *hOGG1* are associated to a larger susceptibility to the total, direct and oxidative, damage to the DNA confirming their interaction in Basic Excision Repair (BER) system, the main repair mechanism of DNA damage including oxidative one.

These findings confirm the role of genetic polymorphisms of genes involved in oxidative stress and DNA repair as biomarkers of individual susceptibility to xenobiotics.

## Data Availability Statement

The raw data supporting the conclusions of this article will be made available by the authors, without undue reservation.

## Ethics Statement

The studies involving human participants were reviewed and approved by ASL (Local Sanitary Agency) of Region Marche. The patients/participants provided their written informed consent to participate in this study.

## Author Contributions

RS: conceptualization and data analysis. BB and DCar: subject enrollment and questionnaire administration. GT, MG, DCar, DP, MG, and NL'E: data collecting. GT, DP, and EP: VOCs' metabolites chemical analysis and DNA and RNA oxidation products analysis. MG and AG: VOCs chemical analysis as airborne and urinary concentrations. DCav, CU, AF, AC, and RM: genotoxicity and oxidative DNA damage biomarkers. PCh and PCa: polymorphisms analysis. RS, PCh, PCa, and DCav: manuscript writing. All authors reviewed and approved the manuscript.

## Conflict of Interest

The authors declare that the research was conducted in the absence of any commercial or financial relationships that could be construed as a potential conflict of interest.

## Abbreviations

VOCs: Volatile organic compounds
OELs: Occupational Exposure Limits
BEI: Biological Exposure Index
SPMA: S-Phenylmercapturic acid
SBMA: S-Benzylmercapturic acid
MHIPPs: Methylhippuric acids
PGA: Phenylglyoxylic acid
MA: Mandelic acid.
